# Insights into the mechanism of the formation of noble metal nanoparticles by *in situ* NMR spectroscopy†

**DOI:** 10.1039/d0na00159g

**Published:** 2020-08-12

**Authors:** Jose Miguel Mateo, Antonio de la Hoz, Laura Usón, Manuel Arruebo, Victor Sebastian, M. Victoria Gomez

**Affiliations:** Department of Inorganic, Organic and Biochemistry, Faculty of Chemical Sciences and Technologies, Universidad de Castilla-La Mancha (UCLM) Av. Camilo José Cela 10 13071 Ciudad Real Spain mariavictoria.gomez@uclm.es; Department of Chemical & Environmental Engineering, Nanoscience Institute of Aragon (INA), Aragón Materials Science Institute, ICMA, University of Zaragoza Mariano Esquillor edif. I+D 50018 Zaragoza Spain; CIBER de Bioingeniería, Biomateriales y Nanomedicina (CIBER-BBN), Centro de Investigación Biomédica en Red C/Monforte de Lemos 3-5, Pabellón 11 28029 Madrid Spain; Regional Institute of Applied Scientific Research (IRICA), Universidad de Castilla-La Mancha (UCLM) Av. Camilo José Cela, sn 13071 Ciudad Real Spain

## Abstract

High-resolution solution Nuclear Magnetic Resonance (NMR) spectroscopy has been used to gain insights into the mechanism of the formation of gold, platinum and gold–platinum alloyed nanoparticles using metal precursors and tetrakis(hydroxymethyl)phosphonium chloride (THPC) as starting materials. THPC is widely used in nanochemistry as a reductant and stabilizer of nanoparticles, however the identity of the species responsible for each role is unknown. The multinuclear study of the reaction media by NMR spectroscopy allowed us to elucidate the structure of all the compounds that participate in the transformation from the metal salt precursor to the reduced metal that forms the nanoparticle, thus clarifying the controversy found in the literature regarding the formation of THPC-based compounds. The progress of the reaction was monitored from the initial moments of the synthesis to the end of the reaction and after long periods of time. Insights into the dual role of THPC were gained, identifying methanol and hydrogen as the actual reducing agents, and tris(hydroxymethyl)phosphine oxide (THPO) as the real stabilizing agent. Finally, the different stabilities of gold and platinum nanoparticles can be attributed to the different catalytic activities of the metals.

## Abstract 2

High-resolution solution NMR spectroscopy has been used to gain insights into the mechanism of the formation of gold, platinum and gold–platinum alloyed nanoparticles using metal precursors and tetrakis(hydroxymethyl)phosphonium chloride (THPC) as starting materials.

## Graphical abstract

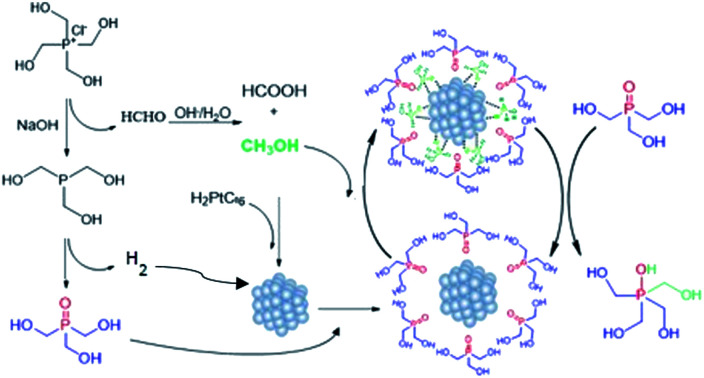

## Introduction

Noble metal nanoparticles are very attractive materials due to their fascinating properties and applications in a wide variety of fields, such as catalysis,^1–4^ biosensing,^5^ water purification^6^ and cancer therapy.^7–9^ In order to control the properties of these materials and open the window to potential applications, it is important to understand the nanoparticle structure as well as its formation mechanism. Numerous approaches have been explored and developed with the aim of generating metal nanoparticles with controlled size and shape. However, molecular mechanisms for the formation of those nanoparticles are not widely explored.

The chemical reduction of metal salt precursors is a widely used strategy to obtain metallic nanostructures.^10–16^ In this approach a reducing agent is mixed with a metal precursor salt in the presence of stabilizing agents such as ligands, polymers or surfactants, which prevent agglomeration of the resulting nanoparticles by electrostatic stabilization or by steric hindrance due to the coupling of large molecules.

A novel method was proposed for the synthesis of gold nanoparticles at room temperature, and consisted in using tetrakis(hydroxymethyl)phosphonium chloride [P^+^(CH_2_OH)_4_Cl^−^] (THPC) as the starting material, an organophosphorus compound which plays a dual role as the reducing and ionic stabilizing agent.^17–23^ THPC finds numerous applications not only in nanochemistry but also in the textile industry, oil industry, leather industry, in medical uses as an oxygen-scavenger, and as a biocide in water systems among others, with 2021 becoming the 100 year anniversary of hydroxymethyl phosphonium salts.^24^ Regarding nanochemistry, the use of this compound resulted in an efficient method to produce nanomaterials with narrow size distribution and high colloidal stability. THPC promotes the nucleation and growth of noble metal atoms and also reduces the surface energy to provide stable ultra-small colloidal nanoparticles. Nevertheless, neither the mechanism of formation of nanoparticles nor the identity of stabilizing and reducing agents has been previously investigated regarding the wide use of THPC for the synthesis of noble metal nanoparticles.

Following this method, Sebastian *et al.* synthesized monometallic nanoparticles and bi-/tri-metallic nanoalloys containing noble metals.^25^ THPC was used as the starting material for the reduction of metal precursors, resulting in nanoparticles which had mean diameters of less than 4 nm with narrow size distributions and high stability in aqueous solution for long periods.

Afterwards, they also investigated the formation of bimetallic monodisperse alloys using THPC, and they were able to control the size of metallic nanoparticles (nanoparticle size < 5 nm) by means of preparing them separately with selected capping agents.^26^

More recently, they reported a novel methodology for the continuous production of Pt-based heterogeneous catalysts based on ultra-small noble metal nanoparticles using THPC.^27^ The use of microfluidic reactors enabled the fast and controlled production of Pt nanoparticles (and also, Pt–Pd, Pt–Ru and Pt–Rh alloy nanoparticles) in short residence times (1–5 min) and with a productivity that was twice the one achieved in conventional batch type reactors.

Neutralization of THPC with a base in the absence of metal salt precursors has been studied,^28,29^ but there is some controversy concerning the formation of THPC derived compounds that take part in the reaction pathway. THPC was reported to react with aqueous sodium hydroxide to give tris(hydroxymethyl)phosphine [P(CH_2_OH)_3_](THP) and formaldehyde (HCHO) at room temperature (1):

1 P^+^(CH_2_OH)_4_Cl^−^ + OH^−^ → P(CH_2_OH)_3_ + CH_2_O + H_2_O + Cl^−^

With an excess of base, the reaction with water continues to provide tris(hydroxymethyl)phosphine oxide [O=P(CH_2_OH)_3_] (THPO) and hydrogen^30^(2):

2 P(CH_2_OH)_3_ + H_2_O → O=P(CH_2_OH)_3_ + H_2_

It is also believed that THP in the presence of an aqueous solution of formaldehyde produces both THPO and tetrakis(hydroxymethyl) phosphonium hydroxide [P^+^(CH_2_OH)_4_OH^−^] (THPOH) but THPOH is rapidly converted to THPO^31–33^(3):

3 P(CH_2_OH)_3_ + CH_2_O + H_2_O → P^+^(CH_2_OH)_4_OH^−^ → O=P(CH_2_OH)_3_ + CH_2_O + H_2_

However, other authors have reported that THPO is formed from THPC through THPOH and THP^34–36^(4):

4 P^+^(CH_2_OH)_4_Cl^−^ + H_2_O ⇌ P^+^(CH_2_OH)_4_OH^−^ + HCl

P^+^(CH_2_OH)_4_OH^−^ ⇌ P(CH_2_OH)_3_ + CH_2_O + H_2_OP(CH_2_OH)_3_ + 1/2O_2_ → O=P(CH_2_OH)_3_

It is also reported in the literature that THP and THPO can be neutralized with formaldehyde to produce hemiacetals, such as P(CH_2_OH)_2_(CH_2_OCH_2_OH) or O=P(CH_2_OH)_2_(CH_2_OCH_2_OH).^35^

It can be seen from all these previous studies^24,28–36^ that both the origin and the order of formation of the different THPC derived compounds remain unclear (*i.e.*, THP, THPO, and THPOH). All of these compounds have been studied by ^1^H and ^31^P NMR spectroscopy under certain experimental conditions of pH and temperature, and the reactions shown in (1)–(4) have been postulated to occur under these conditions.^37–39^ However, evidence for the reaction pathway in the presence of noble metal precursors has not been reported to date. Hence, NMR spectroscopy, as an analytical tool for the characterization of all species that participate in the reaction pathway, could address this gap.^40^

**Fig. 1:**
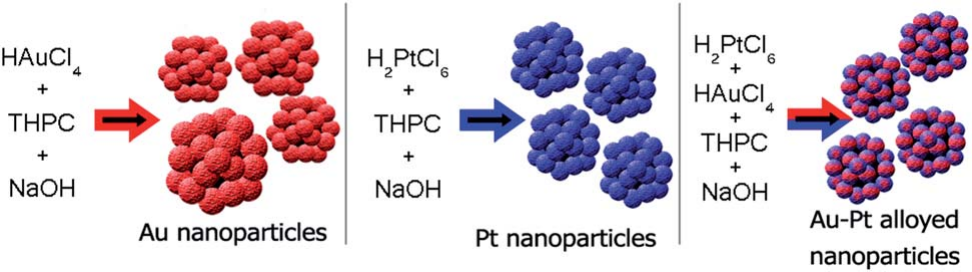
Schematic representation of Au, Pt and Au–Pt nanoparticles synthesized with THPC as the starting material.

We report here a feasible mechanism for the formation of gold and platinum nanoparticles and platinum–gold alloys using tetrakis(hydroxymethyl)phosphonium chloride (THPC) as the starting material (Fig. 1), and employing NMR spectroscopy as an analytical tool.^40^ Using spectroscopic analytical tools, we describe the direct observation of key compounds that participate in the transformation from the metal salt precursor to the reduced form of the metal, from the initial moments of the synthesis to the end of the reaction once the nanoparticles are formed. All the THPC derived compounds that were present in the reaction media were characterized by ^1^H-, ^31^P- and ^13^C-NMR spectroscopy (Fig. S1, S2, S3, Table S1†). Furthermore, evidence for the origin and the order of formation of the THPC derived compounds in the absence and presence of noble metal precursors is presented, with a clear explanation of the formation of THPOH. We explain the different behaviours observed for two different metals (Au and Pt) considering their different catalytic activities, and we also demonstrate by NMR spectroscopy the real role of THPC not only in the reduction of metal salt precursors but also in the stabilization of the resulting nanoparticles.

## Results and discussion

### Control experiments

With the aim of investigating the reaction pathway for the formation of the nanoparticles, several control experiments were carried out and monitored by *in situ*^1^H-NMR spectroscopy (see the Experimental section) from the very beginning of the reaction up to 16 h for Pt and 24 h for Au in order to know the progress of different species present in the reaction media when different reagents were added to the mixture. Hence, three control experiments were performed: (1) THPC in D_2_O, (2) THPC + NaOH in D_2_O and (3) THPC + Pt precursor in D_2_O (Fig. S1†). All these compounds were also characterized by ^31^P NMR and ^13^C NMR spectroscopy (Table S1, Fig. S2 and S3†).

The first control experiment (1) showed a large signal corresponding to THPC (4.61 ppm) along with two signals for THPO (4.07 ppm) and THP (4.00 ppm) as minor components (Fig. S1a†). The molar ratio of the different species in solution is 0.91, 0.045, and 0.045, for THPC, THP and THPO, respectively. THPOH (3.61 ppm) was barely detected in this control experiment. When NaOH was added to the THPC solution, control experiment (2), the initial moments of the reaction show that THPC completely disappeared and the molar ratios were 0.80 and 0.20 for THP and THPO, respectively (Fig. S1b†). For the third control experiment (3), the Pt precursor was added to a solution of THPC in D_2_O (in the absence of NaOH) (Fig. S1c†), and only THPC and THPO were present in the reaction showing a molar ratio of 0.95 and 0.05 for THPC and THPO respectively.

**Fig. 2:**
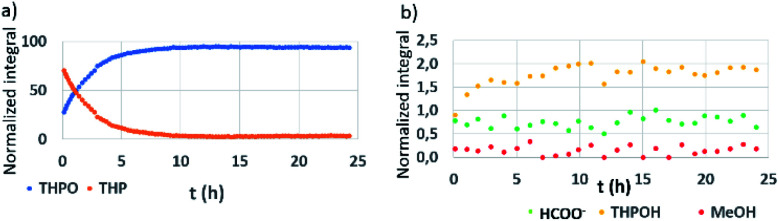
NMR reaction monitoring of THPC/NaOH in D_2_O. (a) THPO (blue) and THP (orange). (b) THPOH (yellow), HCOO^−^ (green), and CH_3_OH (red).

The *in situ*^1^H NMR analysis of the second control experiment (2), when adding NaOH to THPC in D_2_O, is shown in Fig. 2: it could be clearly observed that THPC was totally converted into THP in the initial stages of the reaction (THPC was not observed at time = 0), as the integral value for THP considerably increased when comparing to the integral values in the control experiment numbered as 1 (where THP was almost undetected). The consumption of THP matched to the formation of THPO and THPOH. From 10 h on, THP disappeared from the reaction medium, thus converting itself into THPO (Fig. 2a) as well as into THPOH (Fig. 2b), and then both remained constant with time. Additionally, two other signals appeared with time (Fig. 2b): formate (HCOO^−^)^41^ and CH_3_OH, both compounds coming as a consequence of the release of HCHO from THPC as previously reported.^28,29,36^ It should be taken into account that hydration and dimerization reactions occur when formaldehyde is in an aqueous solution, and the corresponding reaction products are not easily observed by NMR spectroscopy requiring specific methods for their monitoring.^42^ In the absence of basic media, a peak at 4.9 ppm has been observed which might correspond to methylene glycol (HOCH_2_OH), the major specie as a result of a fast hydration of formaldehyde.^42^

**Fig. 3:**
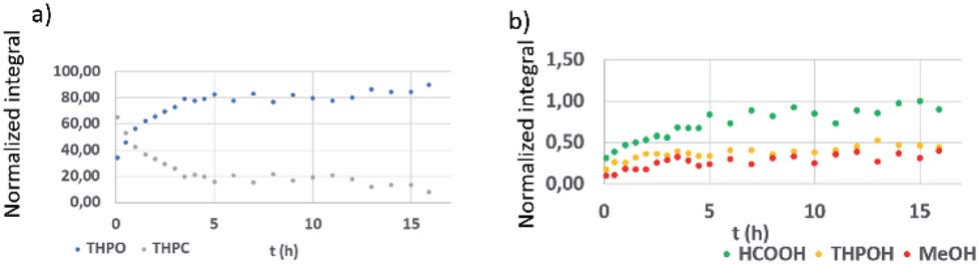
NMR reaction monitoring of THPC/Pt precursor in D_2_O. (a) THPO (blue) and THPC (grey). (b) THPOH (yellow), HCOOH (green), and CH_3_OH (red).

Regarding the *in situ* NMR monitoring of the third control experiment (3) (Pt precursor added to THPC in D_2_O, in the absence of NaOH), the reaction progress is shown in Fig. 3. In the initial stages of the reaction, THPC was present in high amounts and started to decrease gradually (Fig. 3a). Formic acid (8.22 ppm) is identified at a different chemical shift than formate (8.44 ppm) from the previous control experiment, due to the absence of any base in the reaction medium. No evidence of THP was observed, presumably due to the coordination of this compound to the Pt-complex as explained as follows.^43–45^ It is reported that THP is an excellent ligand for platinum. The addition of THP to a solution of PtCl_6_^2−^ renders a platinum complex [PtCl_2_{P(CH_2_OH)_3_}_2_] that can evolve into a complex mixture of platinum coordinated complexes,^44,45^ some of them can undergo intermolecular phosphine exchange rapidly on the NMR time scale and therefore can be observed as broad peaks at room temperature, or even not visible due to relaxation problems by chemical shift anisotropy.^45^ Remarkably, a peak at 4.5 ppm is observed in the control experiment numbered as (3) where the Pt precursor is present (Fig. S1c†), this peak could be attributable to platinum–THP coordinated complexes according to the literature.^45^ This fact can explain the disappearance of THPC in the presence of the Pt precursor while no other THPC derived compound appears over time. The THP that is formed from THPC with time coordinates to Pt and its signal is not visible in the NMR spectrum. While THPC gradually decreases in 5 h (Fig. 3a), THPO (Fig. 3a) and THPOH (Fig. 3b) show a slight increase with time. HCOOH and CH_3_OH were also formed.

From these control experiments, some evidence can already be concluded: THPC is converted into THP, and subsequently, into THPO and THPOH (Fig. 2). Besides, the presence of NaOH accelerates the transformation from THPC to THP, as the former (THPC) is not observed even at the very first reaction stages (Fig. 2a). This fact is also corroborated when looking at Fig. 3a (absence of NaOH), where THPC is still observed. In the presence of the Pt precursor, no evidence of THP is observed, and THPC is being consumed while other THPC derived compounds (THPO or THPOH) have reached the equilibrium concentration (Fig. 3).

### THPC derived compounds in solution for the formation of Pt nanoparticles

**Fig. 4:**
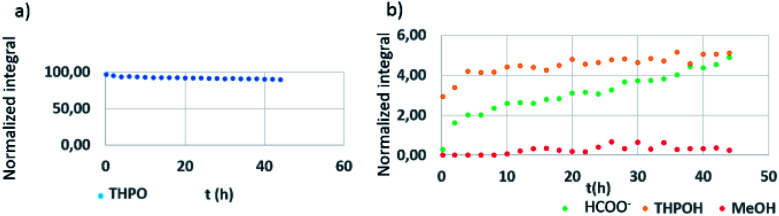
NMR reaction monitoring of THPC/NaOH/Pt precursor in D_2_O. (a) THPO (blue). (b) THPOH (yellow), HCOO^−^ (green), and CH_3_OH (red).

The next step was to monitor the changes produced in the THPC derived compounds during the formation of nanoparticles, by *in situ* NMR monitoring, that is, when simultaneously adding NaOH and the Pt precursor to the THPC solution (Fig. 4). Importantly, as expected, no evidence of THPC and THP was observed during these experiments because of the transformation of THPC into THP in the presence of NaOH, and then into THPO in the presence of the Pt precursor, as likewise observed in the control experiments (Fig. 2a and 3a, respectively). The conversion into THPO was very high at the beginning of the reaction and then slowly decreased with time (Fig. 4a). In contrast, THPOH continuously increased with time, as well as HCOOH and CH_3_OH, the former to a higher extent than the latter (Fig. 4b). An ANOVA T2 Tamahane test (*p* < 0.05) corroborated the trends for all these compounds (Table S2†).

Additional off-line experiments, that is, conducting the reaction in a glass vial and subsequently taking aliquots and analysing them by NMR, showed the same tendency previously observed with the *in situ* NMR reaction monitoring (Table S3†). It has to be mentioned that from 24–48 h on, approximately, the reaction mixture turned from colourless into blackish, the moment at which the nucleation and growth steps of the nanoparticles started.

### THPC derived compounds in solution for the formation of Au nanoparticles and Au–Pt nanoalloys

The differences in the stabilization of the different noble metal nanoparticles (Pt, Au, Rh, and Ru) observed by Sebastian *et al.*^25,26^ aimed to study the reaction for the Au precursor in order to identify possible analogies with the Pt precursor. In the case of Au nanoparticles, different nanostructures with a relatively wide size distribution were observed and showed a tendency to coalesce (Fig. S4†).

**Fig. 5:**
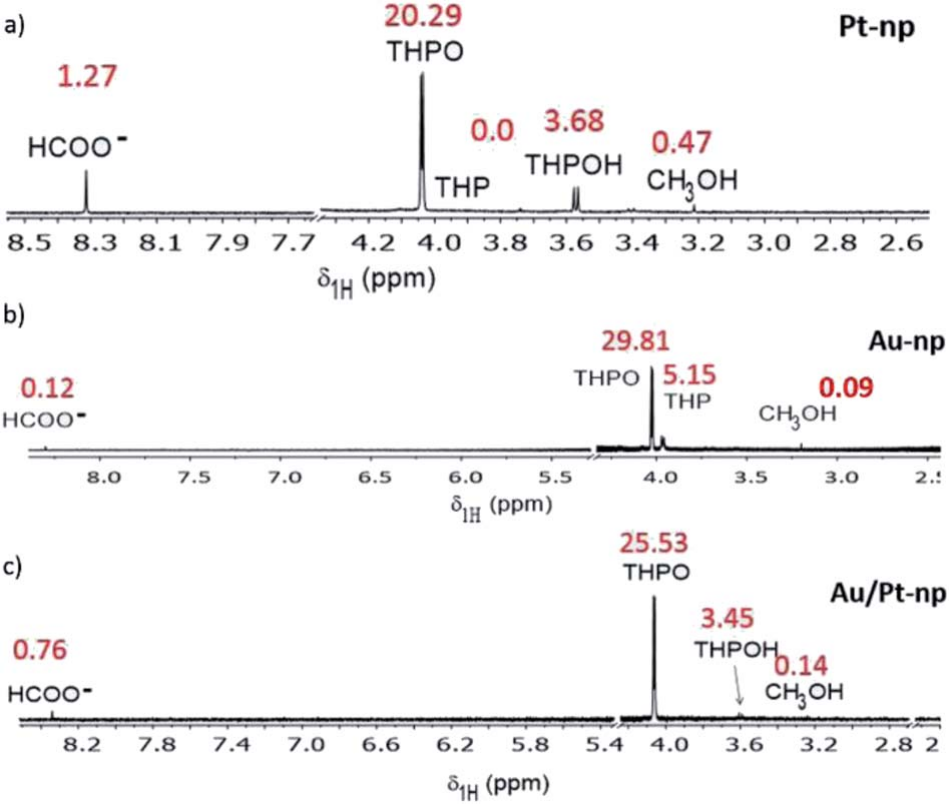
^1^H NMR spectrum for long reaction times (96 h) for (a) Pt nanoparticles, (b) Au nanoparticles, and (c) Au–Pt nanoparticles. Integral values are denoted in red close to every peak. A peak in minor proportion, at 3.4 ppm (in (a)) is visible, however due to its low intensity, it could not be identified.

In order to understand the different behaviour in comparison to the Pt precursor and to evaluate the reaction mechanism, the NMR reaction monitoring for the formation of Au nanoparticles and Au–Pt nanoalloys was also performed. Table S4† shows the evolution with time of the THPC derived compounds that appeared in each reaction, and Fig. 5 summarizes all these observations in the NMR spectra of the different nanoparticles for long reaction times (96 h). In the case of Au nanoparticles, THP was observed in high amounts at the beginning of the reaction and slowly evolved to THPO. THPO increased and THPOH was not observed (Table S4,†Fig. 5). For Au–Pt nanoalloys, a similar behaviour to Pt nanoparticles was observed, but to a minor extent; that is, THPO increased during the first 24 h and its integral value fluctuated showing a very slight decrease with time, and THPOH continuously increased (Table S4,†Fig. 5). In the case of Pt nanoparticles (Table S4,†Fig. 4, 5), THPO was observed at high concentration at the beginning of the reaction and showed a slight decreasing tendency with time, whereas THPOH was present in low amounts and then began to rise slowly (Fig. 4, Tables S3 and S4†). For all cases, HCOO^−^ and MeOH were observed, predominantly in the case of Pt nanoparticles (Fig. 5).

### Mechanism of the formation of noble metal nanoparticles

**Fig. 6:**
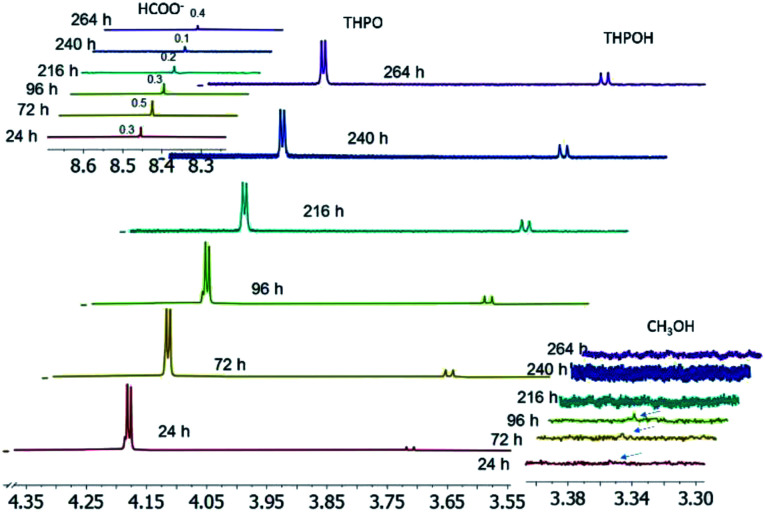
Collection of ^1^H NMR spectra to show the evolution during the formation of Pt nanoparticles. HCOO^−^ (8.45 ppm), THPO (4.18 ppm), CH_3_OH (3.358 ppm), and THPOH (3.71 ppm). Chemical shifts were referenced to TMS.

Once the THPC derived compounds were characterized and the differences between metal nanoparticles were studied, a visual representation of the reaction progress over time to gain insight into the mechanism of this reaction for Pt nanoparticles is shown in Fig. 6. During the course of the reaction, a high concentration of THPO was observed at the beginning of the reaction due to the presence of the Pt precursor that accelerated its formation as mentioned above, and then decreased steadily with reaction time, while the amount of THPOH was very small at the beginning and increased with time, as also shown in Fig. 4. CH_3_OH disappeared with time and HCOO^−^ remained constant (the fluctuation in its integral values is due to the error in the measurement because of the low amount).

At this point, the origin of THPOH during the formation of Pt nanoparticles could be further clarified. In the literature, THPOH has been reported to come from THPC^34–36^ or from THP^31–33^ in the absence of metals. However, in this study a continuous increase in THPOH with time was observed when neither THPC nor THP but THPO was present in the reaction medium as can be observed in Fig. S6.†

**Fig. 7:**
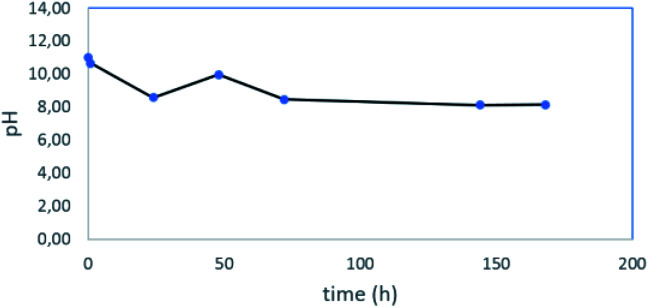
pH values for different reaction times during the synthesis of Pt nanoparticles.

Considering the presence of the different THPC derived compounds when the pH of the THPC solution is varied,^38^ pH measurements were performed with time (Fig. 7). When NaOH and the Pt precursor were added to the THPC solution, the pH increased up to 11 due to the presence of NaOH (THPO was the only THPC derived compound present in the reaction mixture). Once the nanoparticles started to appear, the pH decreased to 8 (THPO and THPOH were in solution). Finally, during the stabilization process the pH slightly increased above 8. At this point, we can conclude that THPOH must be produced from THPO instead of being formed as a consequence of a pH change, since only THPO was present in the reaction medium (Fig. 6, S6†), in contrast to that observed in the control experiments performed in the absence of NaOH where THPOH is formed from THPC (Fig. 3).^38^

**Scheme 1:**
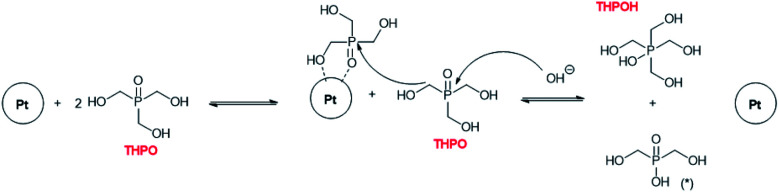
THPOH formation from two equivalents of THPO. The compound marked with (*) was not detected. The product (*) could continue reacting to form sodium phosphate (*δ*_31P_ = 0.00 ppm), which was detected in some cases especially for very long reaction times. The resulting phosphonic acid would be deprotonated by the NaOH present in the medium. Note that this process represents the formation of THPOH when methanol had already been consumed in the reaction medium (see below), corresponding to Fig. S5,† slope b; and Fig. 6, from 96 hours.

Two different zones can be distinguished in Fig. 6, before and after the consumption of methanol. When plotting the ratio of THPOH to THPO integral values *versus* time of data extracted from Fig. 6 (see Fig. S5†), two zones (a and b) with different slopes could be observed within the reaction time (Fig. S5†): zone a surprisingly had a lower slope than zone b and corresponded to reaction times when there was still CH_3_OH in the reaction medium, whereas zone b had a higher slope and corresponded to reaction times when CH_3_OH had already disappeared (Fig. 6). Presumably, this fact might be explained by the coordination of THPO by Pt, as shown in Scheme 1, thus representing a source of *CH_2_OH for the formation of THPOH from THPO.^45^ In this process two equivalents of THPO are required to form one equivalent of THPOH, justifying the more marked decrease of THPO compared to the increase of THPOH (Fig. 6). Therefore Scheme 1 should represent what is happening in zone b (long reaction times). Moreover, compound marked as (*) (Scheme 1) can continue reacting to form sodium phosphate which in fact, has been detected in the ^31^P NMR experiments for long reaction times.

The process shown in Scheme 1 can only take place for Pt but not for Au nanoparticles since, as in the latter case no evidence of THPOH was observed and THPO continued to increase with time (Table S4†).

**Scheme 2:**
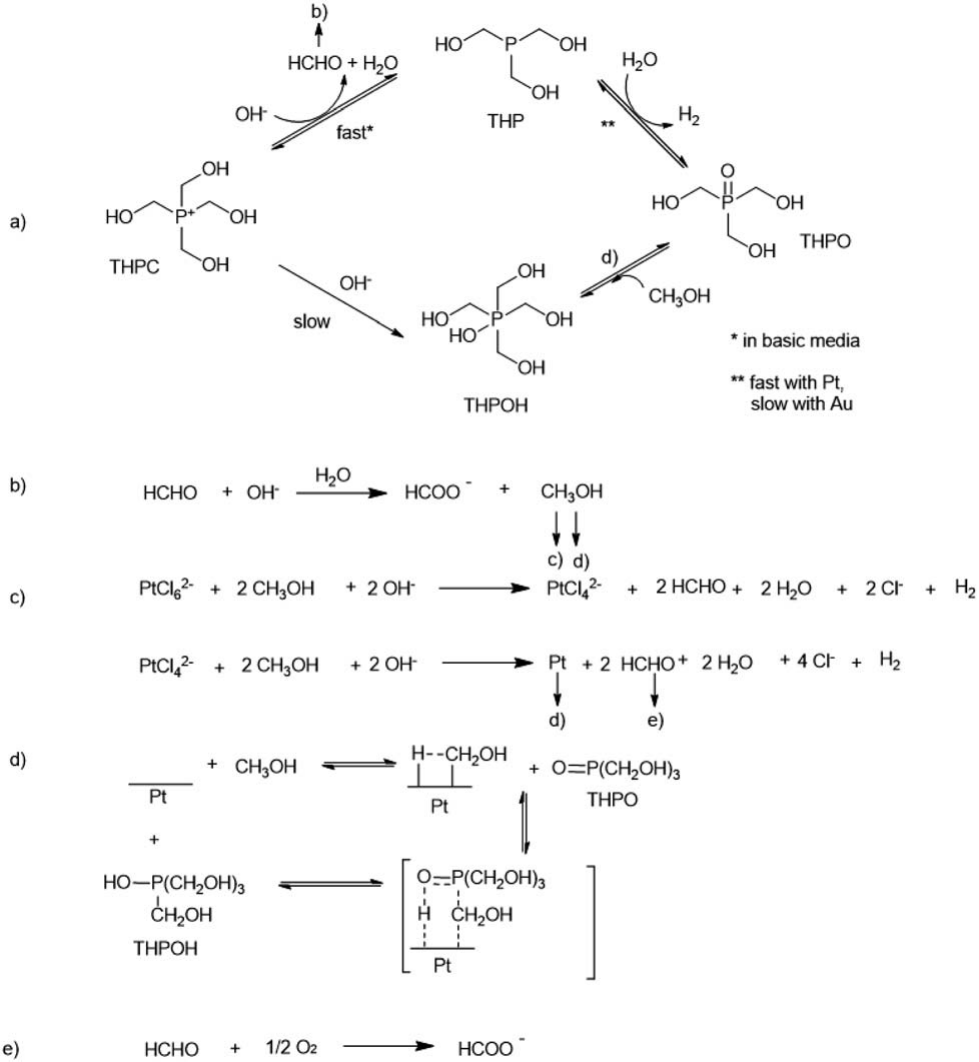
Proposed mechanism for the formation of Pt nanoparticles. (a) Evolution of THPC in the presence of NaOH. (b) Cannizzaro reaction of formaldehyde. (c) Formation of Pt nanoparticles. (d) Formation of THPOH from THPO and methanol. Importantly, the reaction of THPC to THPOH occurs but only to a low extent. Most of the THPOH comes from THPO due to the activation of the C–H bond by the Pt nanoparticles (step d), as reported.^48^ In the case of Au nanoparticles, step d cannot be considered (see text below). (e) Additional formation of HCOO^−^ from HCHO.

Given all the evidence observed from NMR experiments and pH measurements, it is possible to clarify the origin of the different THPC derived compounds and propose that the mechanism of the formation of noble metal nanoparticles using THPC as a starting material is as follows (Scheme 2): as observed during the control experiments, THPC in water is transformed into THP and subsequently into THPO, as also previously reported in certain cases.^24,28–39,46^ The presence of NaOH increases the rate of conversion of THPC to THP because THPC is not observed when NaOH is added (Fig. 2). When the Pt precursor is added to the reaction mixture (Fig. 3), THP is not observed at the very beginning of the reaction, in contrast to THPC which is observed in high amounts. These two facts indicate that THPC is converted to THP (and this is coordinated to the Pt(0)-complex, thus explaining its absence), releasing one equivalent of HCHO, and then THP forms THPO and releases one equivalent of H_2_ (Scheme 2),^24^ a reaction catalysed by the presence of the Pt precursor (Fig. 3). It should be noted that in the excess of NaOH, THPC can be directly transformed into THPO.^47^

Formaldehyde formed in step a undergoes a Cannizzaro reaction, favoured in alkaline medium, to give methanol and formate (Scheme 2b).^49^ A two-step mechanism for the formation of Pt(0) (Scheme 2c) is proposed. In the first step, methanol that formed in step b reduces Pt^4+^ to Pt^2+^, which is corroborated by the yellowish colour of the dispersion.^50^ In the second step, methanol continues to reduce Pt^2+^ to Pt^0^ (black colour of the dispersion) in view of its propensity to form formaldehyde.^51^ Four equivalents of methanol are required to produce one equivalent of Pt(0). Similarly, previous studies also reported that methanol is the reducing agent in the formation of nanoparticles.^52,53^ The formation of THPO generates hydrogen, as evidenced by the pressure release when the reaction vial was opened, and also water, as indicated by the increase in the corresponding signal in the ^1^H NMR spectrum. Note that hydrogen could also participate as a reducing agent according to the literature,^54^ however it is difficult to estimate the contribution of each reducing agent. Formaldehyde can be easily oxidized to formate due to the presence of oxygen in the medium (step e). This fact justifies that formate does not decrease during the reaction (Fig. 4), and methanol completely disappears.

We postulate that methanol also participates in the formation of THPOH from THPO (step d). This can be explained by the activation of the C–H bond of methanol by platinum as Kandoi *et al.*^48^ and others reported. Kandoi *et al.* described all reaction schemes that take place for methanol decomposition on platinum. The first step of the most predominant pathway starts with C–H bond scission (CH_3_OH → *CH_2_OH). This new specie can react with THPO rendering a small amount of THPOH formed during the reaction (Scheme 2, d) which explains the slight decrease of THPO (Fig. 4, 6) and also contributes to the consumption of methanol within time (Fig. 6). Interestingly, the fact that the integral values of methanol plus THPOH match approximately the integral value of formate (Fig. 3b and Table S3†) could corroborate the formation of THPOH from the reaction of THPO with methanol, if we consider that both methanol and formate have been formed from formaldehyde.

This process (THPO to THPOH) is in equilibrium, which explains why the decrease of THPO does not match exactly with the increase of THPOH and that the integral of THPO fluctuates with time, in addition THPOH can also be formed slowly from THPC as mentioned previously.^38^

Note that formaldehyde (4 eq.) is formed in the reduction of PtCl_6_^2−^ to Pt(0) (Scheme 2c) which under our experimental conditions can undergo again the Cannizzaro reaction rendering methanol and formate, justifying the fact that the latter keeps increasing in contrast to when there is an absence of platinum in the reaction medium where the amounts of formate and methanol remain constant with time (Fig. 4b*vs.*2b). The higher absolute amount of formate compared to that of methanol (Fig. 2b) could be due to another process that may take place, the oxidation of formaldehyde in air as the reactions are not carried out in an inert atmosphere.

The mechanism of the formation of Au nanoparticles involves the slow conversion of THPC to THP and then to THPO. At the beginning of the reaction THPC is not observed due to the presence of NaOH. On the contrary, THP and THPO are observed, the former with higher amounts (Table S4†). One possible reason for this could be that THP tends to coordinate to Au(i) to form complexes that are kinetically inert in water.^55^ In addition, no evidence of THPOH was found in these experiments, a fact that may be attributed to the different catalytic activity of Au compared to Pt in the activation of C–H bonds. However, the high catalytic activity of Pt for the activation of C–H and C–C bonds is very well known and makes the activation of the C–H bond of methanol possible.^56,57^

### THPO as a stabilizing agent for the nanoparticles

At this point it is necessary to focus the attention on the lower, integral value of THPO for Pt-nanoparticles and for Pt–Au nanoparticles, compared to Au nanoparticles. These results might be interpreted as THPO acting as the stabilizing agent for the Pt nanoparticles, in a similar way to trioctylphosphine oxide (TOPO), a well-known stabilizing agent, due to the dipolar phosphorus–oxygen bond, which binds to different species such as quantum dots.^58^ The low integral value is justified since the value for a proton in close proximity to a metal nanoparticle is diminished because of relaxation phenomena, as we observed in our previous studies.^59,60^ This role of THPO as the stabilizing agent explains the tendency of Au nanoparticles to coalesce (Fig. S4†), a situation that is in contrast to Pt nanoparticles (and Au–Pt nanoalloys) where the integral values for THPO are much lower due to its stabilizing role (Fig. 5, Table S4†).^59,60^ The tendency of Au nanoparticles to coalesce can also be explained by the different catalytic activity of Au *versus* Pt in the activation of C–H bonds,^54,55^ since THPOH was not observed in the reaction medium when the Au precursor was used, and THPOH is formed as a consequence of the activation of a C–H bond from methanol by platinum. Therefore, step d shown in Scheme 2 is not favoured in the case of Au nanoparticles.

Considering the large excess of THPC compared to the Pt precursor under our experimental conditions, the observed THPO signal at 4.07 ppm should correspond to free THPO molecules in solution instead of THPO-stabilized nanoparticles. An indirect proof of the presence of the nanoparticles is clearly given by the low integral values of THPO in the solution, as explained above.

## Experimental

### Materials and methods

#### NMR measurements

NMR spectra were recorded in D_2_O at 298 K on a Varian Unity INOVA 500 MHz (11.7 T) spectrometer at 499.77 MHz for ^1^H NMR and on a Varian VNMRS 400 MHz (9.4 T) spectrometer at 399.77, 161.82 and 100.53 MHz for ^1^H, ^31^P and ^13^C NMR, respectively. Chemical shifts were referenced to the solvent peak (D_2_O, 4.67 ppm) for the ^1^H NMR experiments. ^13^C NMR and ^31^P NMR spectra were referenced using the internal calibration of the spectrometer based on the solvent. 3-(Trimethylsilyl)propionic-2,2,3,3-d_4_ acid sodium salt (TSP, 0.00 ppm) was used as the internal standard for quantitative ^1^H NMR measurements. Standard pulse sequences from VNMRJ 3.2A software were used for 1D and 2D experiments. The spectra were manually phased and baseline corrected. All spectra were Fourier transformed with MestReNova 10.1 software.

Aliquots from the reaction mixture were extracted at different reaction times, transferred to an NMR tube and then analysed by NMR spectroscopy. Samples for longer periods of time (*t* > 72 h) were analysed from the NMR tube extracted at the end of the reaction (*t* = 72 h). *In situ* NMR reaction monitoring was carried out in a 5 mm-NMR tube inside the NMR magnet. The stoichiometric amount of reagents for these experiments was calculated for the reaction volume of 500 μL (standard volume required for NMR measurements). Several ^1^H NMR experiments were consecutively recorded with the aim of continuously monitor the changes produced during the transformation of the THPC derived compounds and the formation of the nanoparticles.

#### TEM measurements

Preliminary electron microscopy observations were carried out at LMA-INA-UNIZAR using a T20-FEI microscope with a LaB6 electron source fitted with a “SuperTwin®” objective lens allowing a point-to-point resolution of 2.4 Å. HAADF-STEM images were obtained using an aberration corrected scanning transmission electron microscope. Images were acquired using a high angle annular dark field detector in a FEI XFEG TITAN electron microscope operated at 300 kV, equipped with a CETCOR Cs-probe corrector from CEOS Company allowing formation of an electron probe of 0.08 nm.

### Chemicals

Tetrakis(hydroxymethyl)phosphonium chloride solution (THPC, 80% wt Aldrich), hexachloroplatinic acid 8% wt solution (H_2_PtCl_6_, Aldrich), tetrachloroauric acid trihydrate (HAuCl_4_·3H_2_O, Aldrich), sodium hydroxide (NaOH, Aldrich), deuterium oxide (D_2_O, Merck) and 3-(trimethylsilyl)propionic-2,2,3,3-d_4_ acid sodium salt (TSP, Aldrich) were all used as received.

### Synthesis of noble metal nanoparticles

#### Synthesis of platinum nanoparticles

For the synthesis of Pt nanoparticles, 111 μL of a 1 M NaOH solution (111 μmol) was added to 10 mL of D_2_O in a glass vial under magnetic stirring (400 rpm). Afterwards, 67 μL of H_2_PtCl_6_ 8 wt% solution (0.013 μmol) was added to the mixture. After several minutes, 222 μL of a 65 mM THPC solution (14.43 μmol, prepared by adding 12 μL of THPC solution to 1 mL of D_2_O) was added to promote the reaction. The reaction mixture was kept stirring at room temperature for 3 days.

#### Synthesis of gold nanoparticles

For the synthesis of Au nanoparticles, 111 μL of a 1 M NaOH solution (111 μmol) was added to 10 mL of D_2_O in a glass vial under magnetic stirring (400 rpm). Afterwards, 2 mg of HAuCl_4_ solution (5.88 μmol) was added to the mixture. After several minutes, 222 μL of a 65 mM THPC solution (14.43 μmol, prepared by adding 12 μL of THPC solution to 1 mL of D_2_O) was added to promote the reaction. The reaction mixture was kept stirring at room temperature for 3 days.

### Synthesis of gold–platinum nanoalloys

For the synthesis of Au–Pt nanoalloys, 111 μL of a 1 M NaOH solution (111 μmol) was added to 10 mL of D_2_O in a glass vial under magnetic stirring (400 rpm). Afterwards, 33.5 μL of H_2_PtCl_6_ 8 wt% solution (0.0065 μmol) and 1 mg of HAuCl_4_ solution (2.94 μmol) were added to the mixture. After several minutes, 222 μL of a 65 mM THPC solution (14.43 μmol, prepared by adding 12 μL of THPC solution to 1 mL of D_2_O) was added to promote the reaction. The reaction mixture was kept stirring at room temperature for 3 days.

## Conclusions

NMR spectroscopy has provided useful insights for understanding the mechanism of the formation of noble metal nanoparticles, which can be very useful for future studies focused on controlling the particle size and properties of new functional nanomaterials and, in consequence, to improve the formation of metal nanoparticles. Hence, the mechanism of the formation of Au and Pt nanoparticles using THPC as the starting material has been followed by NMR spectroscopy. Spectroscopic evidence for the formation of different THPC derived compounds in basic aqueous media and the pathway in the presence of noble metal precursors enabled the elucidation of the complete mechanism. The participation of CH_3_OH, formed in the Cannizzaro reaction of HCHO, is important during the formation of the metal nanoparticles, as CH_3_OH is the real reducing agent and responsible for the formation of THPOH from THPO. Finally, the lower integral value for THPO observed for Pt nanoparticles compared to Au nanoparticles supports that THPO is the stabilizing agent for the Pt nanoparticles and explains the fact that Au nanoparticles tend to coalesce whilst Pt and Au–Pt nanoalloys are monodisperse.

## Conflicts of interest

There are no conflicts to declare.

## Supplementary Material

NA-002-D0NA00159G-s001
