# Supplementary material for: Insights into the mechanism of the formation of noble metal nanoparticles by in situ NMR spectroscopy
Source: Nanoscale Adv. 2020 Aug 12;2(9):3954–62. doi: 10.1039/d0na00159g (PMC9417889; doi:10.1039/d0na00159g)
Supplement: NA-002-D0NA00159G-s001 [file NA-002-D0NA00159G-s001.pdf]

Electronic Supplementary Material (ESI) for Nanoscale Advances.
This journal is © The Royal Society of Chemistry 2020

SUPPORTING INFORMATION

Insights into the Mechanism of the Formation of Noble Metal Nanoparticles by in situ NMR Spectroscopy

Jose Miguel Mateo,[a] Antonio de la Hoz,[a] Laura Usón,[b,c] Manuel Arruebo,[b,c] Victor Sebastian[b,c] and M. Victoria Gomez*,[a,d]

a. Department of Inorganic, Organic and Biochemistry, Faculty of Chemical Sciences and Technologies, University of Castilla-La Mancha (UCLM), Av. Camilo José Cela 10, 13071 Ciudad Real, Spain.

b. Department of Chemical & Environmental Engineering, Nanoscience Institute of Aragon (INA) & Aragón Materials Science Institute, ICMA, University of Zaragoza, Mariano Esquillor edif. I+D, 50018 Zaragoza, Spain.

c. CIBER de Bioingeniería, Biomateriales y Nanomedicina (CIBER-BBN), Centro de Investigación Biomédica en Red, C/Monforte de Lemos 3-5, Pabellón 11, 28029 Madrid, Spain.

d. Regional Institute of Applied Scientific Research (IRICA), University of Castilla-La Mancha (UCLM), Av. Camilo José Cela, sn, 13071 Ciudad Real, Spain.

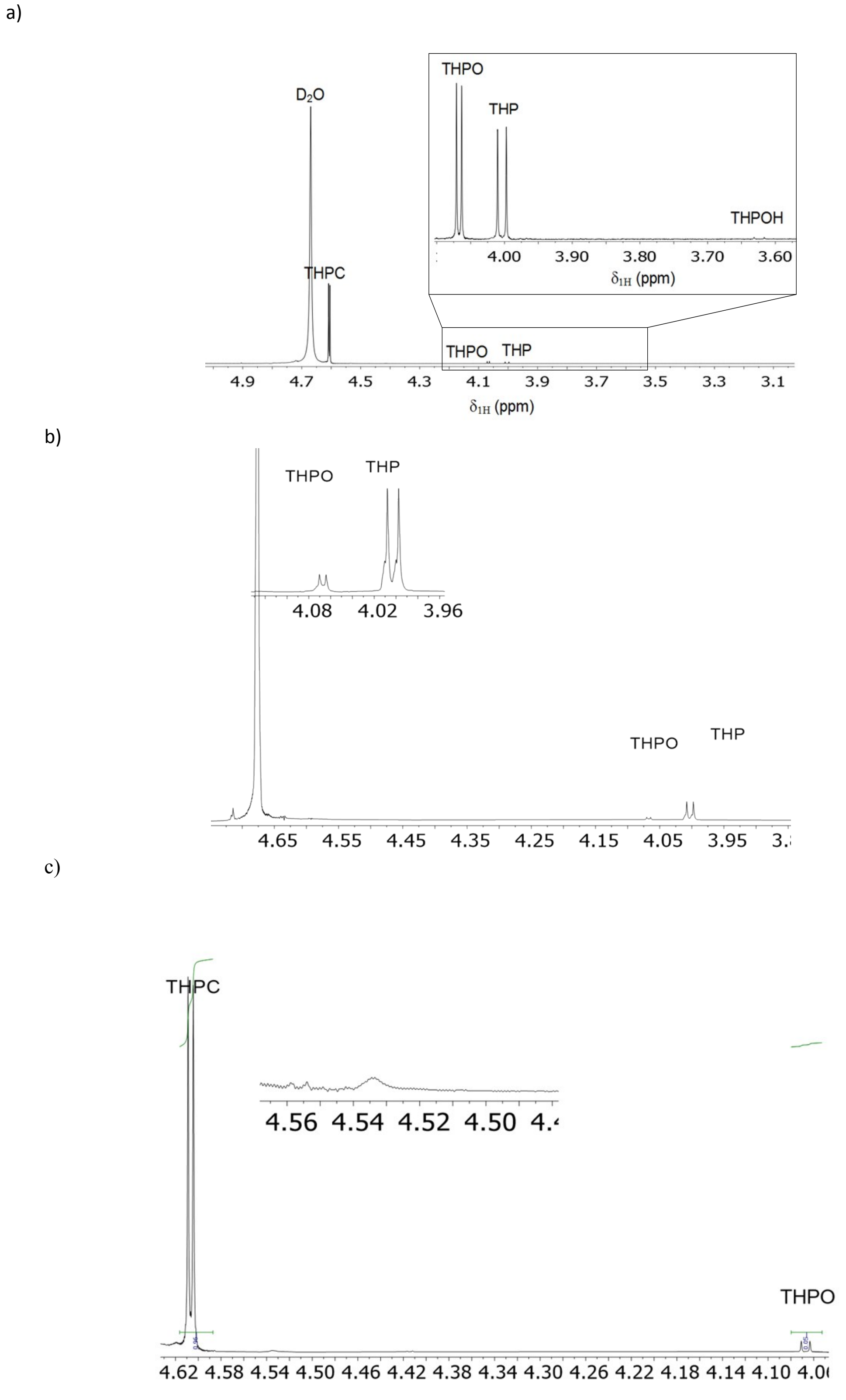

Fig. S1. ^{1}H-NMR chemical shifts of THPC derived compounds. Control experiments for the initial moments of the reaction. a) THPC in D_2O: Molar ratio: 0.91 THPC; 0.045 THPO; 0.045 THP. The insert represents an expansion to show the minor compounds. b) THPC/NaOH in D_2O: Molar ratio: 0.80 THP; 0.20 THPO. Shim misadjustments could be the reason of the observed resolution in the NMR peaks due to the difficulties in the B_0 adjustments at the initial moments of the reaction. c) THPC/Pt precursor in D_2O: Molar ratio: 0.95 THPC; 0.05 THPO. The broad peak at 4.5 ppm shown in the insert could be attributable to a THP-Pt complex not visible in NMR at our experimental conditions (see main text for further explanations). Chemical shifts of the different species: THPC (4.61 ppm), THPO (4.07 ppm), THP (4.00 ppm), THPOH (3.61 ppm).

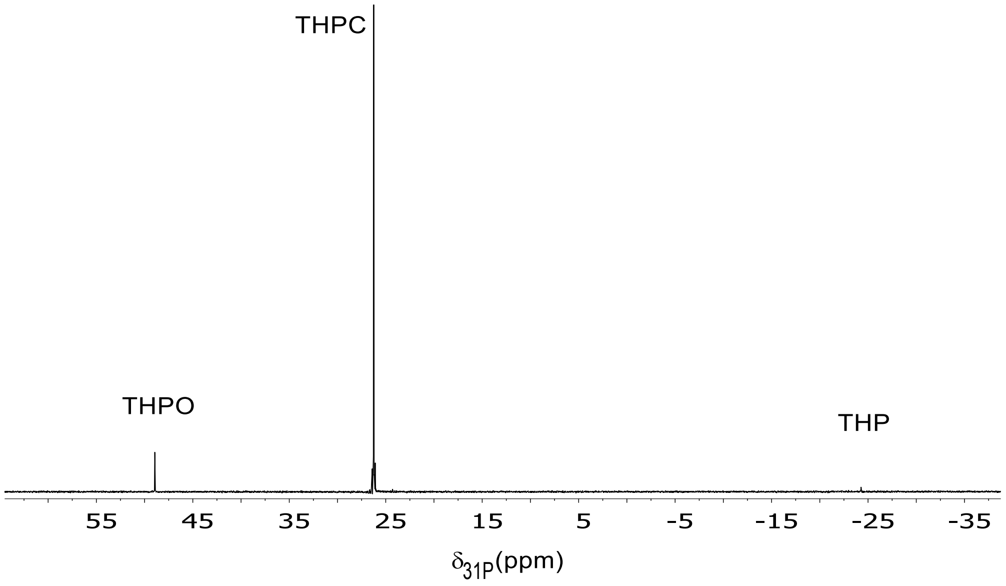

a)

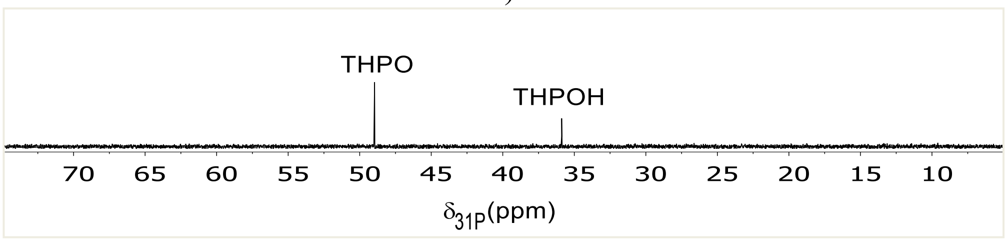

b)

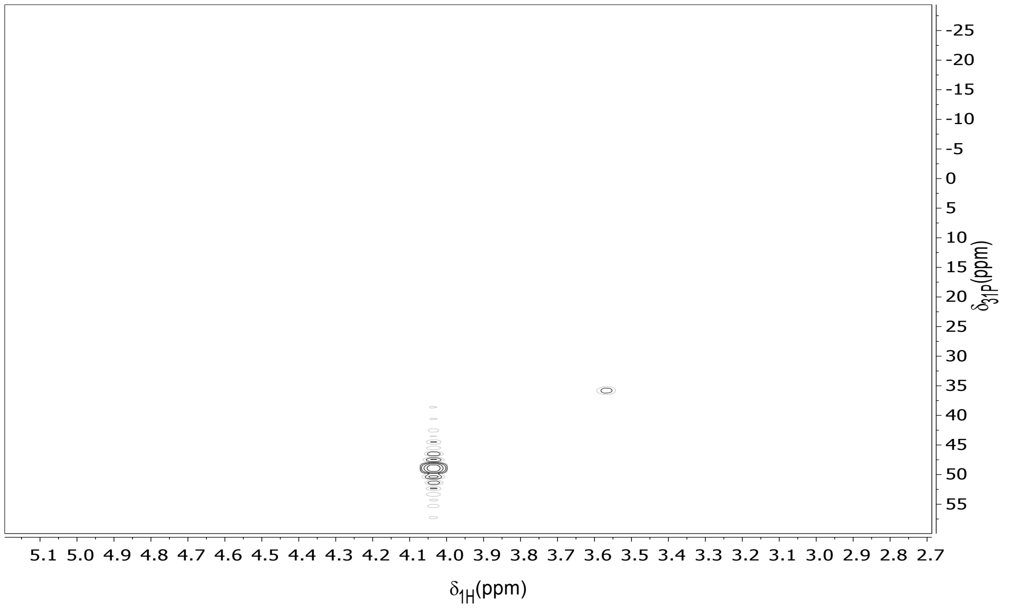

c)

Fig. S2. ^{31}P-NMR chemical shifts of THPC derived compounds. a) THPC in D_2O. b) THPC/NaOH with Pt precursor in D_2O. c) ^{1}H,^{31}P-HMBC of b) (^{1}H,^{31}P correlations of THPC and THP not shown). THPO (48.9 ppm), THPOH (35.8 ppm), THPC (26.3 ppm), THP (-23.8 ppm).

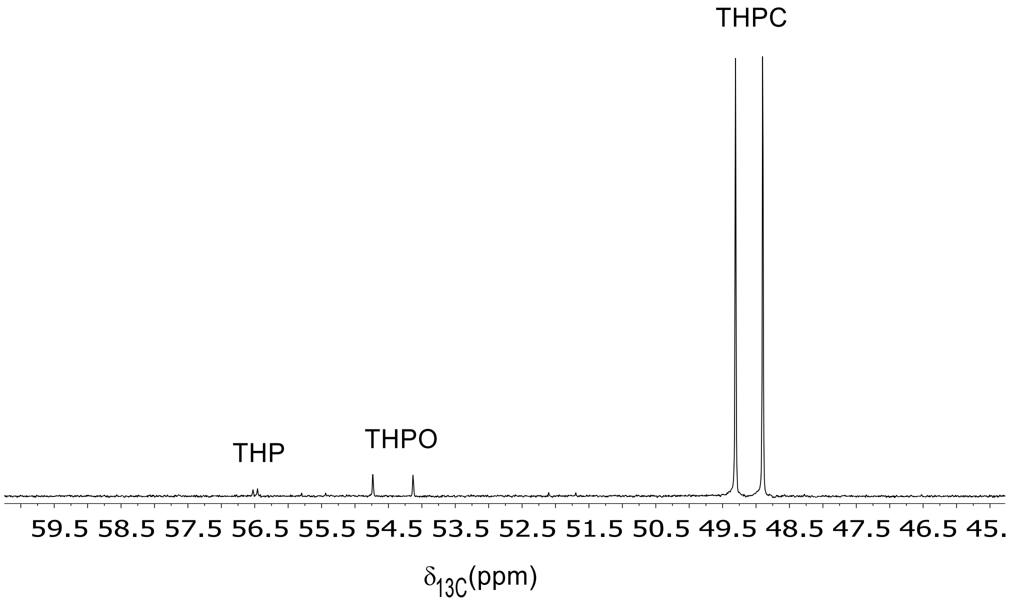

Fig. S3. ^{13}C-NMR chemical shifts of THPC derived compounds. THPC (49.1 ppm), THPO (54.1 ppm), THP (56.4 ppm), THPOH (57.3 ppm). The $^{1}J_{P-C}$ (Hz) is clearly visible (see Table S1 for numerical values). Note two doublets (55.8 ppm and 51.8 ppm) at very low proportions which can be visible at the ^{13}C-NMR spectrum. Attempts to observe the corresponding ^{1}H signals at 2D ^{1}H,^{13}C-NMR spectra were unsuccessful

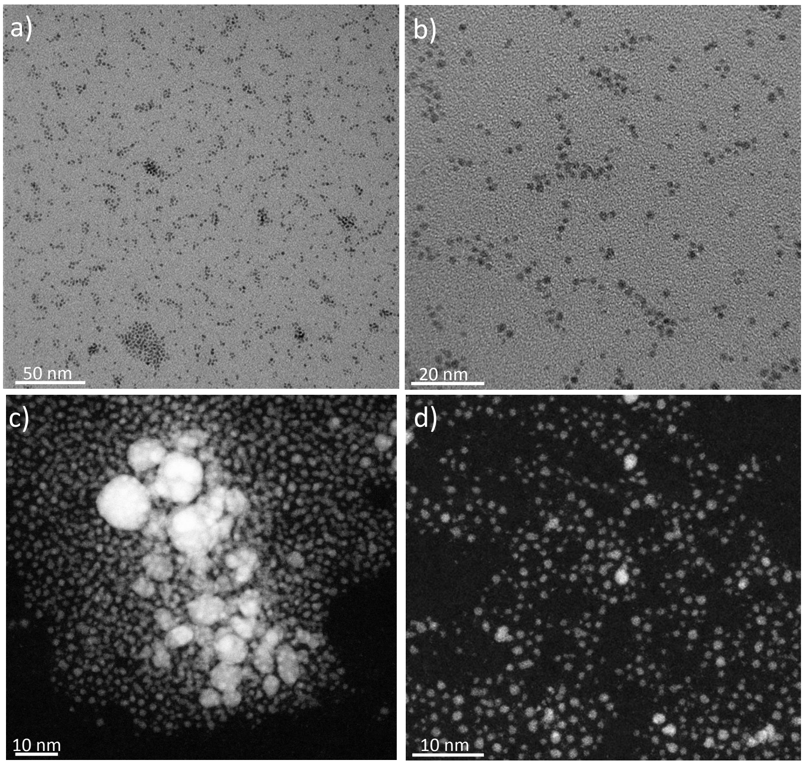

Fig. S4 Electron Microscopy images of nanoparticles produced in D_2O, using THPC at 25 ºC and a synthesis time of 4 days. **a-b)** TEM image of monodisperse Pt nanoparticles with a particle size of 1.8 ± 0.2 nm. **c)** HAADF-STEM- image of Au NPs. **d)** HAADF-STEM image of Au-Pt NPs.

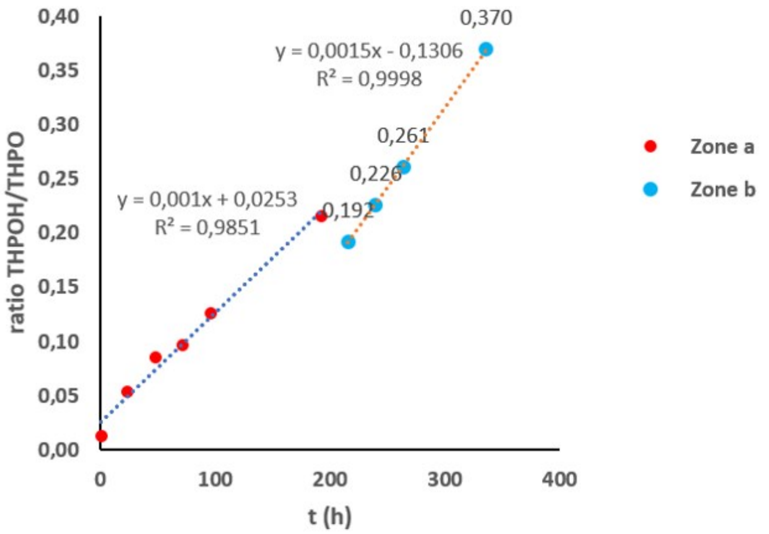

Fig. S5. THPOH/THPO ratio during the formation of the nanoparticles. The red zone corresponds to *zone a* and the blue zone to *zone b*. The ratio *slope zone b/slope zone a* is 1.5. The ^{1}H-NMR spectrum for 216 hours does not show the methanol signal any longer.

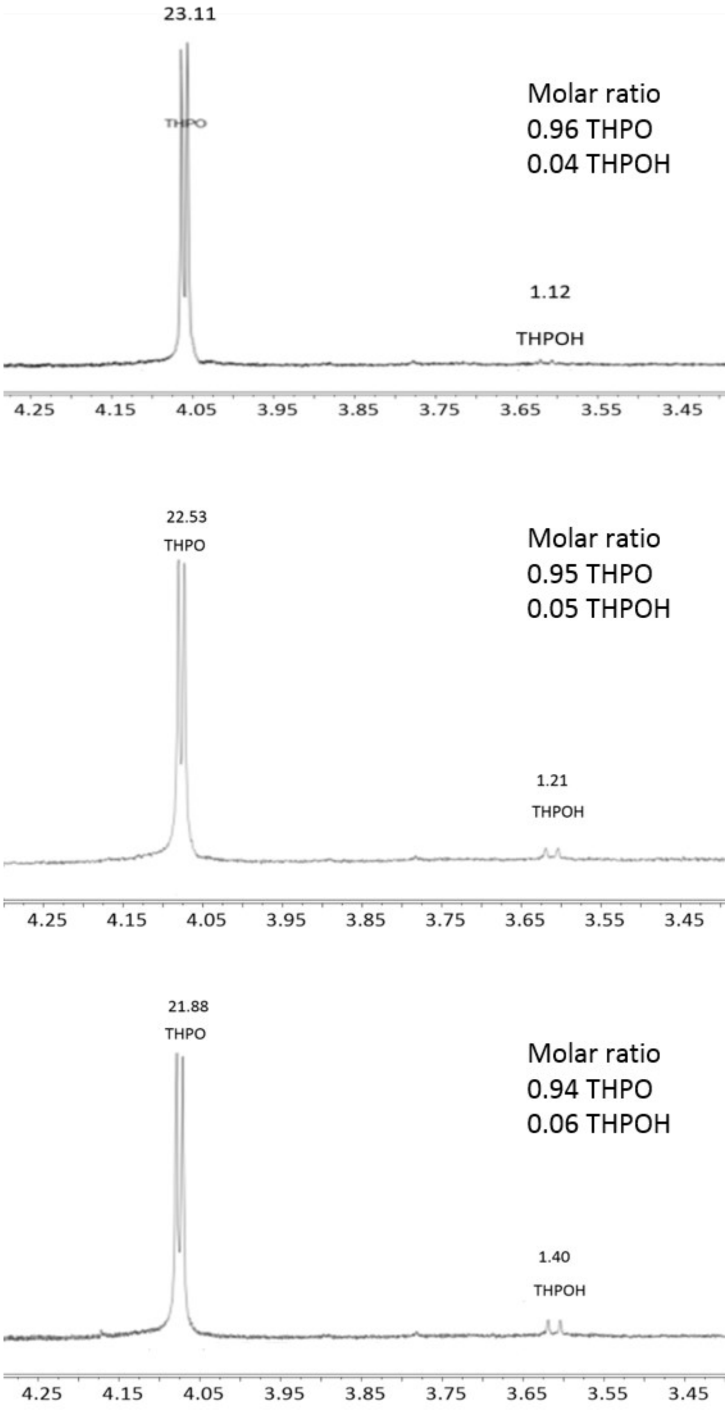

Fig. S6. ^{1}H-NMR spectra within time of a mixture of THPO and THPOH in the presence of nanoparticles: Top) at the beginning of the reaction (t = 0), Middle) after 4 hours and Bottom) after 17 hours. The integral values (THPO/THPOH), referenced to TSP (added as internal standard, integral value of 100), are included in the figure, on top of every peak. It illustrates that THPOH is formed from THPO as indicated in Scheme 1 and 2 (main text).

Table S1. Chemical shifts and coupling constants for THPC derived compounds, referenced to peak solvent (D_2O: 4.67 ppm).

	THPC	THPO	THP	THPOH*
**δ_{1H} (ppm)**	4.61	4.07	4.00	3.61/3.58
**δ_{31P} (ppm)**	26.3	48.9	-23.8	35.8
**δ_{13C} (ppm)**	49.1	54.1	56.4	57.3
**$^{2}J_{P,H}$ (Hz)**	1.8	3.1	5.2	6.2/8.2
**$^{1}J_{P,C}$ (Hz)**	51.0	75.8	7.9	107.4

*It should be mentioned that we have observed, in some cases, a peak signal at 3.58 ppm ($^{2}J_{P,H}$ = 8.2 Hz) that corresponds to Berry pseudo-rotation mechanism of THPOH. Some molecules with trigonal bipyramidal geometry can isomerize by exchanging the two axial ligands for two of the equatorial ones.

Table S2. Statistical analysis by using ANOVA T2 Tamahane´s test ($p < 0.05$) (N = 6-8) for the Pt, Au, Au-Pt and control sample experiments.

	THPO	THP	THPOH
**Pt**	Constant	Not observed	Continuous increase
**Au**	24 h rising, then constant	Slow decrease	Not observed
**Au-Pt**	Constant	Not observed	24 h rising, then constant[b]
**Control sample[a]**	48 h rising, then constant	Continuous decrease	Not observed[b]

[a]Sample in the presence of NaOH and absence of Pt

[b]The intensity was too low to be quantified precisely.

Table S3. Integral values for the formation of Pt nanoparticles (NaOH in the medium) monitored by *off-line* NMR (average value of three samples).

t (h)	$HCOO^-$	THPO	THPOH	CH_3OH
1	0.40	18.87	0.41	0.05
24	1.61	17.96	0.94	1.37
48	4.23	20.02	2.12	1.55
72	3.84	17.25	2.36	1.33

Table S4. *Off-line* NMR analysis for the formation of Pt, Au and Au-Pt nanoparticles, prepared under the same conditions.

	Pt NPs*		Au-Pt nanoalloys*		Au NPs**	
**t (h)**	**THPO**	**THPOH**	**THPO**	**THPOH**	**THPO**	**THP**
**1**	27.27	0.35	20.14	0.22	11.44	40.37
**24**	30.79	1.67	26.89	2.59	21.39	38.68
**96**	26.18	3.31	24.85	3.86	38.91	15.16
**216**	27.53	5.28	25.51	4.13	51.98	0.14
**336**	17.95	6.65	22.40	5.24	55.32	0.00

*No THP observed

**No THPOH observed
